# Back to basics: A re-evaluation of the relevance of imprinting in the genesis of Bowlby’s attachment theory

**DOI:** 10.3389/fpsyg.2022.1033746

**Published:** 2022-12-20

**Authors:** Juan-Pablo Robledo, Ian Cross, Luisa Boada-Bayona, Nadine Demogeot

**Affiliations:** ^1^Millennium Institute for Care Research (MICARE), Santiago, Chile; ^2^Laboratoire INTERPSY, Département de Psychologie, Université de Lorraine, Nancy, France; ^3^Centre for Music and Science, Faculty of Music, University of Cambridge, Cambridge, United Kingdom; ^4^Centre for Multidisciplinary and Intercultural Inquiry (CMII), Department of Health Humanities, University College London, London, United Kingdom

**Keywords:** attachment theory, imprinting (psychology), human imprinting, psychology, ethology, psychoanalysis, social cognition

## Abstract

Attachment theory is one of the key theoretical constructs that underpin explorations of human bonding, taking its current form in John Bowlby’s amalgamation of ideas from psychoanalysis, developmental psychology and ethology. Such a period of interdisciplinary exchange, and Bowlby’s interest in Lorenz’ concept of imprinting in particular, have been subject to rather historical and biographical studies, leaving a fine-grained theoretical scrutiny of the exact relationship between imprinting and attachment still pending. This paper attempts to remedy such an omission by exploring the relationships between these two constructs. It critically reviews the theories of imprinting in general, of human imprinting in particular, and of attachment; analysis of the links between these processes bring to the foreground the distinction between supra-individual vs. individual aspects of bonding, the relevance of ‘proto-attachment’ phases before ‘proper’ Bowlbyan attachment is attained, and the role of communicative signals during such early phases. The paper outlines potential benefits of considering such elements in the study of early social cognition, particularly in respect of the study of the gaze and the infant-directed communicative register.

## Introduction

Attachment theory is probably the prime contemporary scholarly construct in terms of which human bonding is conceptualized and investigated, with a vast body of literature largely– but not exclusively –focusing on the different attachment styles described by (Ainsworth and Wittig, 1969) and their consequences and application in human life across the lifespan. Indeed, moving beyond Bowlby’s initial focus on childhood, attachment styles (Ainsworth and Wittig, 1969; Main and Solomon, 1990; Bretherton, 2003; Rholes and Simpson, 2004; Pearce, 2009) have been used as a vehicle for studying the whole adult lifespan including early adolescence (Hazan and Zeifman, 1994), adulthood (Lopez and Gormley, 2002) and old age (Karantzas and Simpson, 2015). Similarly, the original focus on attachment to parental figures has been expanded to encompass romantic partners, parents, siblings, children and friends (Doherty and Feeney, 2004), adult relationships (Allen and Land, 1999; Heffernan and Fraley, 2015; Overall et al., 2015) and the ways in which attachment influences parenting styles (Jones et al., 2015; Young et al., 2017). The neuroscience of attachment has also been progressively developed (Insel and Young, 2001; Insel and Fernald, 2004; Montague and Lohrenz, 2007; Coan, 2008; Neumann, 2008; Coan, 2010; Panksepp, 2011; Gillath, 2015; Feldman, 2017). Attachment theory is now central to research and academic agendas within clinical applications (Fonagy and Campbell, 2015), adult psychopathology (Ein-Dor and Doron, 2015), and more recently learning (Luyten et al., 2017a,b) and pedagogy (Csibra and Gergely, 2009).

Part of the theory’s robustness and success can be attributed to Bowlby’s (1982) initial efforts in grounding it on contemporaneous scientific evidence, and in going to considerable lengths to create bridges across disciplines. Bowlby borrowed fundamental ideas from the ethologist Konrad Lorenz— in particular, the theory of imprinting (Lorenz, 1935) —and successfully combined them with psychoanalytic developmental theories of object relating (e.g., Holmes, 1993; Blatt et al., 1997; Wachtel, 2010; Fonagy et al., 2018). This successful amalgamation was also made possible by a propitious period of interdisciplinary open-ness during the 1950s and 60s that counted with many illustrious figures spanning from ethology to psychoanalysis (see “Human imprinting”). Such a period in general, and Bowlby’s interest in Lorenz’ work in particular, have so far been highlighted and approached through partially theoretical, partially historical and biographical scopes (e.g., van der Horst, 2009, 2011; Vicedo, 2009). In a similar fashion, the present paper aims to draw academic attention back to the intellectual roots of Bowlby’s theories by exploring ways in which the theories of imprinting and attachment can be construed as related. Not quite as previous works, however, this article does so not with a historical or biographical focus, but with a precise, theoretical one. Although literature mentioned in the previous paragraph, as well as Bowlby’s (1982) own formulation of attachment theory evidence a significant overlap between said theory and that of human imprinting, Bowlby himself did not provide an explicit, reciprocal positioning of both concepts either despite or perhaps because of their apparent overlap. This article’s simple goal is thus providing a rendering of the relationship between the two constructs that is more explicit and precise than has been so far advanced.

To this end, we trace attachment back to imprinting in three sections. The first section reviews the general theory of imprinting, with an emphasis on the literature available at the time Bowlby hatched his own theory and from which he drew several of his core ideas. The second section delves into a concept that is largely unrepresented in contemporary literature: human imprinting. A natural projection stemming from Lorenz’s initial work on non-human animals, the concept can be thought of as the prequel of attachment theory, and was eventually effectively replaced by the latter in the field of psychology. The third section is then dedicated to Bowlby’s initial formulation of attachment theory, with a particular focus on his account of the mechanisms behind its establishing, an aspect which, as will be argued in the discussion, could have been taken for granted rather than questioned. Thus, the contents in this section will— hopefully —differ from usual accounts in the literature. These three sections are integral to the present paper’s primary contribution: an in-depth analysis of both theories’ core formulations in terms of their overlaps and differences.

Providing a new perspective on the initial formulation of attachment theory and its links to imprinting should, we hope, bring a number of benefits. First, it should provide a higher degree of theoretical clarity than currently exists, useful to anyone interested in the subject. Second, returning to the basics of any theory with a fresh pair of eyes and a warranted skepticism is a healthy exercise that counters the ossification of knowledge and its potential transformation into unquestioned dogma. Although Bowlby certainly delivered a carefully-made theory, there are likely to be assumptions he did not question, and possibilities he dismissed in favor of the ones that made it to the canonical version of attachment theory. Third, the paper revisits a corpus of literature seldom cited today that could be useful to contemporary scholars less familiar with Bowlby’s initial formulation of attachment theory. Fourth and most importantly, some elements of attachment theory— particularly its onset and early stages —that rely the most on imprinting and its focus on concrete mechanisms are likely to better resonate with contemporary empirical research than it could in the 1960s. Indeed, in the context of current research in attachment, in comparative, developmental and cognitive psychology, a revaluation of the roots of attachment theory could spark discussion about the similarities and differences between the different theoretical models and how they can complement each other. In particular, it could be of benefit to the study of early social interaction and cognition (including, for example, the functions of signals such as the gaze and Infant Directed Speech) by affording a reappraisal through the lens of attachment theory. This final subject is further unpacked in the paper’s discussion.

## Imprinting

By the dawn of the 20th century, animal behavior was explained fundamentally through the concept of ‘instinct’. Although Charles Darwin (1871) had convincingly portrayed the construct as a set of inherited, unlearned behavioral patterns of high adaptive value, clear and extensive understanding and description of what it implied was still missing (Van der Horst, 2011). In this context, the idea of imprinting was mainly developed by the biologists Konrad Lorenz and Niko Tinbergen, who observed how young greylag goslings and jackdaws would not recognize members of their own species directly after birth, but show a strong following response to the first moving object in their surroundings (Lorenz, 1935). On the one hand, such a following response seemed to always take place in a relatively fixed manner without being taught. On the other hand, the fact that the object that would elicit such a behavior could vary implied that the behavior itself still partly depended on interaction with the environment. Thus, Lorenz (1935) coined the term ‘imprinting’, and described it as a process by which some species learn to recognize members of their own species, which enables them to become the object of subsequent behavior patterns, including mating. Imprinting largely comprised the idea that early social contact determines the character of adult social behavior.

Central to imprinting is the concept of ‘social releasers’ (or ‘releasers’): particular features of morphology or behavior that elicit instinctual behavior in the organism that perceives them (Lorenz, 1935). In other words, releasers are species-specific signals, and an infant will react and engage in the imprinting process depending on the degree to which the caregiver can supply the proper signals/releasers. The reaction to a specific releaser would be mediated by activity in specific coordinating centers in the infant’s central nervous system Lorenz called ‘innate releasing mechanisms’ (IRM). At a behavioral level, the structured pattern of movements that constitute the reaction itself was conceptualized as a ‘fixed action pattern’ (FAP), which develops without conventional reward such as food. Lorenz portrayed FAPs as highly stereotyped innate movement patterns that would run to completion regardless of further stimulation, thus comprising a concrete rendering of the genetically-programmed core of a species’ typical behavior (Van der Horst, 2011). On the infant’s part, Lorenz introduced the concept of paedomorphic traits (or *Kindchenschema*) as an innate releasing mechanism for caretaking behavior and affective orientation toward infants. Paedomorphic traits consist of protruding cheeks, a large forehead and large eyes below the horizontal midline of the skull. Baby faces showing these features are commonly described as cute or attractive (Sternglanz et al., 1977).

In Lorenz’s approach to his initial observation of goslings, the phenotypic features and caring behavior of geese parents would normally ‘release’ (trigger) in their goslings a FAP, which in this example corresponds to the unlearned response of following. In imprinting studies, the most commonly found FAP was and remains proximity-seeking behavior, which typically takes the form of approaching or clinging to the caregiver. Because of the biologically-set fit between a species’ releasers and FAPs, accidental imprinting to an object different from the biological parents is not likely to happen. Yet, Lorenz (and many other ethologists and animal psychologists after him) demonstrated that despite the rather rigid character of FAPs, goslings and other species infants could be imprinted to human beings and even inanimate objects, evidencing that imprinting also decisively depends on environmental factors. For instance, although the paedomorphic traits of an infant of a given species are fundamentally designed to influence members of its own kind, they can nevertheless be effective in other species and thus prompt imprinting. Through these concepts and evidence, Lorenz connected the internal, innate behavior patterns of animals with external stimuli, demonstrating that imprinting is a process neither instinctive nor learned (Van der Horst, 2011).

There are four distinctive properties that Lorenz (1935) initially attributed to imprinting. First, that it takes place only during a brief ‘critical period’ in the life-cycle of an organism. This means, for instance, that ducklings presented to a given object between 13 and 16 h after hatching would show a consistent following response to that object. The same will not happen, however, if presented before or after that time. The chronological limits of the critical period vary from species to species (and at a finer level, from individual to individual), but the developmental events that seemed to account for its beginning and end are the infant’s capacity to generate the relevant FAP, and the onset of fear of strangers, respectively (Gray, 1958). A second postulated property is that imprinting is irreversible; once fixated to an object, FAPs will be unlikely to be released by a different kind of object. In other words, if first released by a human being during its critical period, a gosling’s following response will be unlikely to be released by a member of a species other than humans. The third property is that imprinting is a form of supra-individual learning. This property will prove very important when distinguishing between imprinting and attachment, which makes it worth explaining in detail. It was previously stated that if an organism (or even an inanimate object) is presented during a gosling’s critical period for imprinting, its features will likely trigger an unlearned response in the gosling. Such features will be learned by the infant, so it can recognize them and react in the future. However, it is not the organism’s individual features (the ones that make it recognizable as a unique entity) that are learned, but generic features of its species. Accordingly, when the mother goose presents itself to the gosling during the latter’s critical period, it is not her individual features as a distinctive organism that the gosling first learns and later recognizes, but rather the general features of her species. The fourth property states that imprinting influences patterns of behavior that have not yet developed in the organism’s repertoire. This idea was already briefly mentioned and it largely rests in the supra-individual nature of imprinting: if a gosling is imprinted by one of its parents, it is geese features as a species that the infant learns, and it will be such species-specific features that will, in the future, trigger its mating behavior. Alternatively, if imprinted to a human being, the target of the goose’s future sexual drive will be human beings, and not geese.

Soon, imprinting was investigated not only in birds, but also in mammals— including human beings —and even in fish and insects (Hess, 1959). Furthermore, the concept remained only briefly restricted to ethological circles and soon elicited great interest in other disciplines, prompting a rapidly growing corpus of interdisciplinary cooperation. Perhaps the most fruitful example of such cooperative projects was the Tavistock seminar series on mother-infant interaction, held between 1959 and 1963. Unsurprisingly, the interdisciplinary discussion held by psychologists, psychiatrists, obstetricians, neuroscientists, psychoanalysts and zoologists raised criticisms regarding Lorenz’s initial postulates about imprinting. Neither the critical period nor the irreversibility or the supra-individual criteria were found to be as clear as originally claimed. For instance, the ethologist Robert Hinde concluded that rather than denoting a dichotomy in development (Hinde, 1963), a critical period must be understood as a change in the probability that a certain learning will take place. Accordingly, the sharpness of the limits of a critical period will depend on the detail of the criteria used in its measurement (e.g., considering the act of ‘following’ as a given species’ proximity-seeking behavior, as opposed to a further operationalized account involving measured distance, time-lapse, and so on). Additionally, the psychologist and psychoanalyst John A. Ambrose observed that each response behavior has its own critical period, rendering the idea of a ‘general’ critical period for imprinting as inaccurate (Ambrose, 1963). Two categories of critical periods were distinguished: a critical period for learning behavior X which is critical for the subsequent performance of X, and a critical period for learning behavior X which is critical for subsequent performance of behavior Y. This idea will be further explained in the next section.

Imprinting research has continued until the present day. The emphasis has been largely on the nature of its underlying mechanisms, including its neuroscience (see Rogers et al., 2013; Montuori and Honey, 2015). The main thrust in imprinting research apart from the emphasis on mechanism has been individual-level recognition, first in recognizing the mother and, second, in setting up a standard against which the individual can prefer to mate with a partner that differs slightly from close kin— recognized as a result of sexual imprinting (for a review see Bateson, 2014). Although much more could be adduced regarding the development of this field, what remains important is to bear in mind the basic principles hereby outlined, as they largely shaped an understanding of the human case.

## Human imprinting

The first comprehensive proposal for human imprinting was made by the American psychologist Philip Gray. Essentially relying on Lorenz’s work, Gray (1958) conceived it as the first form of socialization between the infant and its kind, as in most or all species where a social complex is vital for survival. Although significantly criticized, the proposal contained at least three main ideas that prompted crucial debate regarding human imprinting. Such a debate was held by an international, interdisciplinary network of scientists such as Jean Piaget and Mary Ainsworth, as well as Lorenz and Bowlby themselves, who gathered for decisive events such as the Geneva WHO study group in 1955, or the four “Ciba-symposia” (also referred to as meetings of the Tavistock study group) held in 1959, 1961, 1963 and 1965 (for an in-depth account of these meeting, see van der Horst, 2009). Particular authors and concepts relevant for the present paper are mentioned in this section.

Firstly, Gray proposed that humankind is an animal species whose neonates are unable to manifest any motor equivalent to the following response found in birds. His second claim was that this should have forced the evolution of a different system of releasers and FAPs for human imprinting. Particularly, the smile of human infants could be regarded as an analogous version of the following response found in birds. Thirdly, the critical period for imprinting in humans, Gray proposed, would take place from about 6 weeks to about 6 months, beginning with the onset of learning ability, continuing with the achievement of the smiling response, and ending with the development of fear of strangers.

Gray’s first assertion has proven so far irrefutable. Indeed, our species’ neonates are unable to manifest any motor equivalent to the following response found in birds. As discussed, the proximity-seeking behavior (an important case of FAP) that most avian species studied show as a response to the caregiver’s releasers is the act of following. The degree of precociality of these species implies that motor development of their neonates affords locomotion and thus the act of following. New-born primates, more altricial than the birds but still on the precocial side of the precocial-altricial continuum (Starck, 1998), are mostly incapable of following the carer.

Given that imprinting is a biological and social process essential for the adult sexual behavior of an organism and thus the perpetuation of its species, selective pressures would have favored primate neonates able to generate an FAP other than following, suitable for their motor development and yet equally functional. In this respect, Bowlby (1982) points out that although the instinctive behavior of virtually all members of a species conforms to a common overall plan, the particular form it takes in any one individual is often distinctive, and may in fact be quite unusual. Thus, a bird of a species that habitually nests in trees may nest on cliffs when no trees are available, or a mammal of a species that normally gathers in flocks may be ungregarious if reared away from its kind. These cases, Bowlby argues, illustrate how the development of a behavioral system that appears to be environmentally very stable (as the act of following had so far been) may nonetheless be open to some degree to influence by the environment in which development occurs (Bowlby, 1982).

The selected analogous FAP was clinging. Indeed, at birth or soon after, all primate infants— except humans —cling to their mothers (see Harlow, 1958; Harlow and Harlow, 1962). In lower members of the primate order (e.g., lemurs or marmosets) the infant must, from birth onward, cling without any assistance from its mother. In later species like Old World monkeys the infant actively clings, except in the early days of its life when its mother provides— some —support. In the most advanced apes, gorillas and human beings, clinging is included in the neonate’s behavioral equipment as a reflex, but without the necessary strength to support themselves for long (Bowlby, 1982). By this token, the evolutionary shift from following to clinging as privileged proximity-seeking behavior is largely an adaptation to the selective pressures of altriciality. The human case entails yet a different intersection between motor development and FAPs, as *Homo heidelbergensis* infants faced an even greater challenge in their attempt to secure imprinting: secondary altriciality (Ruff and Walker, 1993). Indeed, human neonates’ immaturity makes them unable to follow or indeed exert any kind of locomotion. Furthermore, as discussed by Falk (2004a), it also prevents them from relying on clinging either, otherwise the privileged proximity-seeking behavior in primates.

The implications of secondary altriciality for human imprinting led to Gray’s second relevant claim, proposing the smile response in human infants as the motor equivalent of the following response in animals below the higher primates. In other words, he proposed the smile as an evolved, analogous FAP. This claim is not self-evident and indeed gave rise to a series of critiques within the academic community. Although Ambrose (1963) criticized Gray’s lack of arguments, he nevertheless acknowledged the suitability of the proposal in the context of human inability for locomotion. Indeed, the notion that the ‘true’ or ‘social’ smile does not appear much before 6 weeks of age had already been suggested by Darwin (1877) and is still validated by contemporary research (Jones et al., 1991). Ambrose and British ethologist Robert Hinde (1963) agreed that the sensitive period for smiling is crucial, not so much specifically for later occurrences of smiling as for the development of human social responsiveness in general (which is a case for the aforementioned pattern of the critical period of behavior *X* being critical for the subsequent performance of behavior *Y*). In other words, it is not only the immediate responses which smiling evokes from the mother that must be taken into consideration, but also the manner in which it influences her bonding experience at a higher level of integration (Hinde, 1963). The smile response, although certainly crucial, remains thus only as an index among all kinds of bodily movements and sounds which stand as attempts to form social interaction.

It is therefore in the multimodal context of bodily activity that supra-individual learning takes place; from birth and up to about four or 6 months of age, a human infant learns the multimodal morphological and communicative characteristics of the species it is been imprinted into, ‘*gradually piecing together and building up his picture of what a human being is like’* (Ambrose, 1963, p.207). The close interrelation of the smile and the rest of the senses was soon confirmed by Freedman’s studies on congenitally blind children (Freedman, 1964). The universal presence of smiling among cultures along with its continued appearance in congenitally blind infants led him to conclude that the facilitation of earlier smiles by a high-pitched voice can be interpreted as an ethological-type releasing mechanism. Therefore, although vision definitely facilitates smiling, no single sensory channel can be claimed as its exclusive releaser. Ambrose (1963) acknowledged that crying— the preeminent manifestation of anxiety or fear in the early months of human infants —works just as effectively as a proximity-seeking behavior as smiling does. Furthermore, he noted that in an important number of cases smiling can take place only once crying has succeeded in achieving proximity and thus ensured the basics for survival. Although Ambrose did not go into greater detail regarding the role of infant cry in human imprinting, as will be addressed in the next sections, his observation proved to be highly relevant for attachment theory and the study of infant cry in general. Thus, although there may not be a privileged FAP in human imprinting, it is clear that neonatal crying and the myriad of behaviors that constitute proto-conversation allow for it to take place. More importantly, the exclusion of following and clinging implies that human imprinting concentrates in communicative behavior like no other mammalian species does.

Gray’s third claim, concerning the limits of the critical period for human imprinting, also gave rise to a series of critiques within the academic community. Regarding the start, Gray held the working hypothesis that in species not born at an advanced stage of neural and motor maturation there is a pre-learning period where the ‘higher’ parts of the brain are deemed immature and thus conditioning would not be possible. Such a claim was soon dismissed by American psychologist Howard Moltz, who argued that imprinting develops rather independently of conventional reward, such as food and thus could not be considered a form of conditioning (Moltz, 1960). Furthermore, Gray’s claim works against an enormous corpus of investigation on infant learning. As an example, recent research shows that newborns’ cry melody evidences vocal learning partially based on the influence of surrounding speech prosody (Mampe et al., 2009). Such learning processes occur during the first 2–5 days of life, in other words, during the ‘pre-learning’ period proposed by Gray.

Since the issue of the role of smiling has already been discussed, what remains of this section will focus on the end phase of human imprinting. American psychologist Eckhard Hess’s early hypothesis that the onset of fear marks the end of the critical period for imprinting seems to have only partially survived further evidence (Hess, 1959). In both human and non-human animal development there is an initial period following birth during which no fear reactions to strangers can be found. This makes imprinting possible in the first place, since fear responses would prevent an infant from engaging in the kind of social behavior necessary for imprinting to take place, avoiding rather than seeking proximity toward a potential imprinting object (Gray, 1958). Conversely, fear of strangers prevents the infant from imprinting onto an endless list of individuals and their corresponding species. The onset of fearful behavior was initially explained in purely maturational terms (Gessel and Thompson, 1934), but rather became considered as a function of the individual’s perceptual experiences; fear in human infants would arise when an object is familiar enough to activate habitual processes of perception whilst at the same time sufficiently dissimilar to arouse incompatible ones, and thereby disrupt the central neural patterns laid down by previous stimulation (Hebb, 1946). Thus, an initial period of experience is a necessary prerequisite in order to establish the notion of the familiar and give rise to the feeling of discrepancy with new patterns of sensory input. German child psychologist H. R. Schaffer added that the fear of strangers relies on the ability to distinguish familiar from unfamiliar individuals, a faculty which develops in the months preceding the onset of fear (Schaffer, 1966). In concrete terms, Schaffer postulated that it is a change in cognitive structure, the establishment of the object concept (Piaget, 1955), that allows the infant to go beyond a stage where it experiences only a series of images which may be recognized, but have no continuity or substance. Through this change in cognitive structure the infant becomes ‘object-oriented’ rather than ‘stimulus-oriented’ (Schaffer, 1966, p.103). It has more recently been argued that what emerges between 5 and 12 months is the ability to *demonstrate* an understanding of object concept, with the understanding itself already having been acquired (Diamond, 1991). Hebb’s and Schaffer’s theories are compatible and underlie the importance of both the supra-individual and individual aspects of imprinting, as well as their interrelation. The supra-individual phase of imprinting, during which the infant learns the multimodal morphological characteristics of the carer’s species, affords the achievement of a sense of the familiar. The infant gradually becomes familiar with stimuli such as features and behavior, but these stimuli would not be associated with any particular subject. Nevertheless, as Ambrose’s (1963) observations on human infants and Harlow’s experiments on non-human primates (Harlow and Harlow, 1962) pointed out, the carer shows more than morphological characteristics; it shows caring behavior, which progressively distinguishes it from just any other member of the group and the species. Such a dyadic process, added to the cognitive development of an object concept, would thus enable the infant to organize familiar features into particular subjects. Consequently, other subjects become unfamiliar and the infant begins to fear them. Bowlby (1982) even argued that it is not only fear itself, but rather the recognition of the carer(s) at an individual level, which sets the end of the imprinting process. He also observed that the stronger the original imprinting, the more persistent the avoidance of anything new. However, if a young animal is forcibly kept in the presence of a new object, the fear response may be partially or wholly habituated. By this token, Lorenz’s initial claim of imprinting as a kind of supra-individual learning has had to be nuanced in order to embrace the parallel development of an individual-specific dimension.

## Attachment theory

By now, any reader familiarized with attachment theory should have a sense of its substantial overlap with that of human imprinting. As it will become obvious after the next sections, these discussions and most of the research program on human imprinting were, in respect of human development, largely replaced by the more psychology-oriented scope of attachment theory. Because readers are probably far more familiar with the latter than the former, this section will be less comprehensive. It will instead focus on Bowlby’s initial formulations, exclusively stemming from the first volume of his classic trilogy, that prove essential for the article’s discussion.

As mentioned in previous sections, mutual influence in the development of imprinting and attachment theories is fairly well documented. Freud had emphasized the central role of child-caregiver early interaction on the former’s adult life— including their sexual behavior —when Lorenz and Tinbergen hatched the idea of imprinting in birds (Vicedo, 2009). Consequently, child analysts from various psychoanalytical schools were at the time the link between an infant’s tie to its mother on their adult personality. In this context, John Bowlby started following Lorenz’s work and gained interest in ethology. Bowlby had amassed a body of observational data, nevertheless, his findings were not yet conclusive, partly due to a lack of experimental examination as well as a comprehensive theoretical framework (Van der Horst, 2011). Lorenz in turn became interested in Bowlby’s research, which largely supported his own ideas.

Bowlby (1982) described attachment roughly as a category of social behavior leading children to maintain proximity to their mother-figure. Attachment behavior comprised two core elements: maintaining or restoring proximity to someone, and the specificity of that someone. As an alternative to the idea of instinct and in line with contemporary ethology, analytical biology, and control theory and cybernetics, Bowlby approached the bond between a child and their mother as the result of several behavioral systems that hold proximity as a predictable goal. The main element that would allow an organism to fulfill such an adaptive, goal-directed system is the idea of feedback. Though borrowed from disciplines beyond the study of organisms, Bowlby considered fit applying such principles to living organisms including human beings, who simply evolved in and for their own ‘environment of evolutionary adaptedness’. Following Hinde’s (1959) ideas and as a substitute for the controversial idea of instinct, any biological character that in its development is little influenced by variations of environment was considered ‘environmentally stable’ (as opposed to ‘labile’). Such an approach applies to above mentioned FAPs such as the clinging, following, or smiling. Similarly, a system’s sensitivity to its environment can fluctuate through time. Thus, Bowlby favored the notion of ‘sensitive periods’ over Lorenz’s original ‘critical periods’.

As previously mentioned, following Bowlby’s definition, an infant’s capability for seeking proximity (the first element) cannot be properly labeled as attachment behavior until it is preferentially aimed at a specific individual (the second element). Regarding the first component he observed that human infants are endowed with a number of behavioral systems out from birth. First, there is a perceptual apparatus that orients the infant toward their caregivers. In addition, effectors such as hands, feet, head and mouth further facilitate interpersonal contact. Thirdly, neonates have what Bowlby considered as ‘signaling equipment’— voicing, neonatal crying, and limb gestures. Such behaviors were deemed essential during the attachment process’ first phase, characterized by orientation and signals with limited discrimination of a figure. Delineated between birth and around eight and 12 weeks of age, in this phase the child’s ability to differentiate individuals is restricted to smell and touch. Typical behavior includes orientation toward people, grasping and reaching, eye tracking, as well as babbling and smiling. As a whole, such behavior is meant to maximize the chances and duration of proximity, by influencing the caregiver.

In theory, a next attachment phase is attained once orientation and signaling stably narrow down toward one or more discriminated individuals, with sensitivity to auditory stimuli being evident earlier than those involving the vision. The upper limit of such a second stage was theoretically set at around6 months of age. By then, two key changes determine a third phase. On the one hand, proximity-seeking visibly narrows down to one individual. On the other hand, major progress in locomotion means that proximity is at last no longer reduced to signals. Behavioral repertoire thus expands to encompass following someone, greeting in return, and using the attachment figure as a base for exploration. Bowlby identified two main reasons behind the specification of proximity-seeking. In an affiliative motion, a consolidation in the knowledge of the individual features of an attachment figure, as opposed to the supra-individual characteristics shared by human beings as a kind. In contrary, phobic motion, fear and withdrawal progressively become a spontaneous reaction to any unknown persons. The connexion between these two complementary processes, or their relative importance or mutual causation, were not discussed by Bowlby. Moreover, roughly between nine and 18 months of life, all above mentioned systems (perception, signaling, locomotion) mediating a toddler’s behavior to their attachment figure become organized on a goal-corrected basis, becomes evident for any spectator. During this final, fourth phase by watching the main caregiver’s behavior and environment, the toddler gradually infers their goals and means for execution, establishing a goal-corrected partnership.

In the initial formulation contained in his trilogy, Bowlby systematically covered the different angles his theory entailed, a relevant one being the affective dimension of attachment. Some essential elements of such dimension are the dyadic regulation processes that lead to the infant’s feeling of ‘perceived safety’ or separation anxiety, their sedimentation into what was later on called attachment styles, as well as their consequences up until old age. However, these elements, most of which were mentioned in the introduction, do not fall into the article’s focus.

In his discussion of the term presented in the first volume of Attachment and Loss, Bowlby initially acknowledged the diverse uses of the term “imprinting” by different disciplines and from different approaches. Bowlby argued that the knowledge of the development of attachment behavior in humans could be summarized briefly under the same heads that describe imprinting in birds. Such an argument rather depicts both constructs as equivalent in the sense of belonging to the same category. Furthermore, after problematizing the convergent evolution of imprinting in birds and mammals, he immediately attributed imprinting’s main characteristics to attachment behavior, tacitly implying an analogous status.

However, after the evidence and arguments presented in the previous sections, it can be posited that Bowlby in fact used the word ‘attachment’ to refer to two intimately related yet distinct phenomena. On the one hand, the word mainly refers to proximity-seeking behavior that is directed toward a discriminated figure and integrated into a goal-corrected system: in other words, ‘proper’ attachment, or attachment in the strict sense. On the other hand, it refers to proximity-seeking behavior that can be found— in human beings, mammals and birds alike —before ‘proper’ attachment has been achieved, somewhere around its third phase. For the sake of clarity, let us henceforth refer to ‘proper’ Bowlbyan attachment simply as ‘attachment’, and to preliminary bonding phenomena as ‘proto-attachment’.

For clarity sake, let us try to accurately position both attachment and proto-attachment behaviors in respect to imprinting. Proto-attachment behavior largely corresponds to the supra-individual phase of imprinting. Any new-born organism that learns the supra-individual features of the carer figure will engage in some kind of proximity-seeking behavior toward members of the latter’s kind. This proximity-seeking behavior can be regarded as a form of proto-attachment, triggered by and directed toward particular releasers (signals): morphology (i.e., general size and shape), particular features (i.e., feather coloring,), acoustic signals (i.e., a loving, soothing voice), etc. ‘Proper’ Bowlbyan attachment is a process that has successful supra-individual and individual learning as prerequisites, but that develops into further distinctive stages which culminate with inherently dyadic and affective dynamics that can be said to be beyond the scope of imprinting theory.

## Discussion

### Implications of the imprinting/attachment scrutiny

Bowlby’s claim that humankind is the slowest species in terms of the attainment of (‘proper’) attachment has at least three implications that will be critically addressed here. These concern the nature of proto-attachment, the exact transition from proto-attachment to attachment, and the role of the infant’s signaling repertoire during proto-attachment.

Regarding the first issue, Bowlby sets the achievement of individual discrimination as a prerequisite for ‘proper’ attachment, whilst at the same time acknowledging that some degree of discrimination is present from the start. Such early discrimination mainly concerns human features: the auditory stimuli that characterize the human voice, visual features of a human face, as well as the tactile and kinaesthetic stimuli proper of human arms and body. Furthermore, he recognized that human stimuli do prompt behavior in the infant that, although not yet integrated into the goal-corrected attachment system, does result in achieving proximity. Hence, in setting individual discrimination as a prerequisite for ‘proper’ attachment, Bowlby discussed but did not further focus on the supra-individual attachment behavior that takes place during the first phase of attachment and is superseded by further sophisticated individual discrimination at some point during the second phase. The notion of supra-individual attachment behavior— or proximity-seeking behavior that is not directed toward discriminated objects but to classes of objects (any human being) —is largely what imprinting originally entailed: a process by which some species learn to recognize members of their own species and direct toward them a series of fundamental behaviors that start with proximity seeking, to later add others such as mating. Bowlby could have further approached supra-individual (proto-)attachment behavior by drawing on the literature on imprinting that explicitly dealt with this issue. However, his research interests and the effect these exerted on his own understanding of imprinting led him in another direction.

The second issue concerns the task of locating the onset of human attachment (as opposed to proto-attachment) through a given behavioral or developmental landmark, or at least within a reasonably delimited period. Bowlby opted for the latter. Whilst acknowledging an inevitable degree of arbitrariness, he still aimed to locate such an onset within one of the four phases of attachment he proposed. An infant would not yet be attached during the first phase, and already attached when entering phase three. Therefore, whether and to what extent an infant can be said to be ‘properly’ attached during the second phase is a matter of how attachment is defined. Unfortunately, Bowlby did not proceed to explicitly deal with the implications of his own definition of attachment on this issue. Nevertheless, by observing that human infants are able to distinguish their mother-figure before they can either cling or move, Bowlby did take a position— even if implicitly. The resulting sequence can be reconstructed as follows: individual discrimination is slowly but firmly acquired from the beginning, motor skills (such as clinging and locomotion) being added only later, and finally both behavioral systems are organized in the goal-corrected system of attachment.

Finally, in any case Bowlby did consider gross (limb) motor skills as a hallmark of the third attachment phase at which he deemed the infant to be already attached, revealing the importance he attributed to such behaviors. Given that in all other studied species proximity-seeking behaviors take the form of some kind of gross motor skill, it is unsurprising that Bowlby did not consider the human case of attachment complete without them. A tacit assumption, therefore, is that all the signaling repertoire (neonatal crying, vocalization, and gestures) that human infants are born with and that quickly develops is not integrated along with the capacity for individual recognition into the full-blown, goal-corrected (‘proper’) attachment system until the mastering of limb motor skills is added. Accordingly, before the rather vaguely-defined moment during the second phase of attachment in which the infant is supposed to ‘become attached’, the infant’s signaling equipment is essentially considered as no more than ‘building-bricks’ for the later development of attachment: a set of behavioral systems to become elaborated and to be superseded by more sophisticated ones, largely non-functional until becoming integrated into a functional whole. In short, although Bowlby did discuss the role of the infant’s signaling repertoire during ‘proto-attachment’, his interest in such mechanisms was restricted and the discussion succinct when compared to the further developed issues of attachment and loss— the true scope of his work. As an illustration, such mechanisms are listed in a single section (“Behavioral equipment of the human neonate”) within a single chapter (chapter 14) among three whole volumes of writing.

These three implications and their stress on the onset stages of attachment (or, ‘proto’ attachment) circle back to the human imprinting debate. As discussed, whilst crucial, an infant’s smile remains merely an index amid a much larger set of bodily movements and sounds which stand as attempts to form social interaction. Contemporary understanding of such ‘bodily movements’ has made significant progress since Bowlby’s time. An infant’s early communicative interaction largely takes the form of (but is not reduced to) what has since then been conceptualized as ‘protoconversation’ (Bråten, 1988; Levinson, 2006). The latter, just as much as later adult conversation, consists of the multimodal integration of ‘interaction engine’: the face-to-face, turn-organised coordination of mutual gaze, body and facial gesture, and vocalization that characterizes human face-to-face communication. Rudimentary elements of protoconversation can be witnessed from birth (largely through imitation), with fully observable deployment by between 6 and 12-weeks of life (Trevarthen, 1993).

As previously mentioned, the study of early signaling was not nearly as developed during Bowlby’s lifetime as it is now, with significant progress since then in various strands of research concerning early social cognition. A first major element to consider in early signaling and interaction is gaze. Notwithstanding the fact that its study has made tremendous progress, especially if compared to the state of the art during Bowlby’s lifetime when eye tracking technology was not available, its link to attachment in general, or its onset in particular, are not widespread and far from completely understood. For instance, although the link between gaze and attachment has been explicitly studied, it has been so in the context of young adults in their twenties (Cecchini et al., 2015; Prinsen et al., 2019). Similarly, even when research has delved into the link between attachment and gaze in children whose age actually corresponds to Bowlby’s four onset phases (Koulomzin et al., 2002), explicit discussion concerning such phases is absent. By this token, an opportunity for advancing the description and mapping of the ‘proto-attachment’ phases, as well their link to early social cognition, is lost.

Closely related to gaze is the issue of joint attention, a phenomenon that emerges as early as 6 months of age (Charman and Charman, 2003) thus also well before the attainment of ‘proper’ attachment. Exploration of the link between such a phenomenon and attachment could also benefit from this paper’s arguments as research assessing the link between joint attention and attachment styles has so far not involved children young enough to correspond to ‘proto-attachment’ phases. More concretely, the earliest study we could find in terms of age involved 12-to 20-month-old children (Mohammadzade Naghashan et al., 2021). In a similar vein, literature addressing joint attention in atypically-developing populations such as toddlers with autism warn that most research on the subject involved participants over 3 years-old (Naber et al., 2007). Such a trend, Naber and collaborators add, overlooks the need to study early precursors of disrupted social behaviors that are essential to understanding how to mitigate or change such deficits in social cognition at later developmental stages. This in turn provides a further example of the lack of attention that ‘proto-attachment’ has received, the potential for correcting such inattention, as well as the corresponding potential clinical benefits.

Shifting from the visual to the aural domain, the link between vocal/acoustic signals and the early stages of attachment is a subject that could bear further strengthening. Modifications to the maternal human pelvis necessary to accommodate bipedalism caused a selective shift toward premature and helpless neonates (Falk, 1998) whose ability to cling actively to their mothers (as is the case in other primates) was accordingly lost. As a result, distal mother-infant gestural communications increased (Tomasello and Camaioni, 1997) and prosodic affective vocalisations became ubiquitous to compensate for the reduction in sustained mother-infant physical contact. Such a shift thus stimulated the development of infant cry on the one hand (Soltis, 2004), and on parental vocal response to it on the other hand, as ‘disembodied extensions of mothers’ cradling arms’ (Falk, 2004b, p. 462). Such paternal vocal response, has been conventionally subdivided into infant-directed speech— also referred to as ‘motherese’ (Fernald, 1985), ‘babytalk’ (Singh et al., 2002) or ‘songese’ (Longhi, 2009) and infant-directed singing (Trehub et al., 1993). Although several researchers have indirectly related infant-directed speech and infant-directed singing to attachment (see Trehub et al., 1997; Swain et al., 2004; Shannon, 2006; Smith and Trainor, 2008), such mentions do not lead to discussion at this level of detail, nor relate motherese to the early stages of attachment (i.e., ‘proto-attachment’) in a specific manner. In this sense, there is scope to render such links more explicit and systematic.

Taken together, examples discussed in these three last paragraphs— gaze, joint attention and parental prosody, respectively —provide proof of concept for the idea that the onset stages of attachment and their underlying, concrete mechanisms entail a microcosm that was less graspable in Bowlby’s time, has become more accessible for contemporary science and methods, and thus should receive scientific attention. Whilst such attention seems to be currently being paid through the scope of social cognition rather than that of attachment theory, both approaches are equally important and legitimate, as well as mutually beneficial. By bringing attention back to such early, ‘proto-attachment’ phases and their underlying mechanisms, the field of attachment theory would not miss a body of interactional phenomena that was originally labeled as ‘imprinting’ and that largely inspired the hatching of attachment theory itself.

A final point of discussion concerns the place ‘proto-attachment’ and human-imprinting-related phenomena occupy in the contemporary body of literature on attachment theory. The present article was largely motivated by the impression that, to our knowledge, in-depth critiques of the theoretical foundations of attachment theory (in general) or of its exact connection to imprinting (in particular) are not many. It could be that, perhaps because of the broad scope of applicability of attachment theory, Bowlby’s initial efforts for studying the establishment of attachment (i.e., his interest in human imprinting and the early ‘proto-attachment’ phases) have not been widely adopted. In this sense, it is interesting to wonder to what extent Bowlby’s theoretical foundations of attachment remain essentially unchallenged by those who adhere to it. Of course, such a suggestion remains a mere hypothesis, yet one that could be tested through a literature review. In the same vein and more concretely, for instance, it could be hypothesized that, should a systematic review on the subject be undertaken, only a handful of exceptions in the literature on attachment would make any mention of human imprinting at all.

## Conclusion

In sum, it can be stated that Bowlbyan attachment takes place only after transitioning from an initial imprinting-like, supra-individual, ‘proto-attachment’ phase, through a series of preferences that further develop. Although attachment and imprinting may to a significant extent refer the same list of phenomena, their scopes of application imply different emphases. On the one hand, imprinting theory stresses a series of mechanisms that enable the very first social interactions of an infant as well as later ones that are eventually crucial. Bowlbyan attachment, on the other hand, stresses the psychological bond inherent in such social dynamics. In other words, imprinting is involved in the attachment of a child to their mother, but those researchers that work on attachment are also interested in the development of an affective relationship. Such a difference in scopes becomes natural when considering the contexts of ethology vs. psychology/psychoanalysis these two constructs came from.

Supra-individual learning of objects and the ‘proto-attachment’ behavior that is directed toward them does not occupy a central role in Bowlby’s definition of both imprinting and ‘proper’ attachment, thus diminishing the chances of researchers taking an interest in and exploring them. Stemming from Bowlby’s proposal, raising the question of the extent to which research in the field of human attachment has reproduced this omission.

Together, these distinctions and subtleties stress the importance of bearing in mind Bowlby’s landmarks in the transition through the early stages of attachment, or ‘proto-attachment’, as well as their use in the study of early social cognition.

## Author contributions

J-PR conceived the article’s main argument, undertook most of the research and literature review, and wrote most of the article. IC provided substantial feedback during the conception of the article, and substantially contributed to the article’s writing. LB-B and ND contributed to the writing of the article’s introduction and discussion. All authors contributed to the article and approved the submitted version.

## Funding

This research was supported by the ANID Millennium Science Initiative Program (ICS2019_024).

## Conflict of interest

The authors declare that the research was conducted in the absence of any commercial or financial relationships that could be construed as a potential conflict of interest.

## Publisher’s note

All claims expressed in this article are solely those of the authors and do not necessarily represent those of their affiliated organizations, or those of the publisher, the editors and the reviewers. Any product that may be evaluated in this article, or claim that may be made by its manufacturer, is not guaranteed or endorsed by the publisher.
